# ZSCAN-DDIE: an interpretable zero-shot learning method for the prediction of drug-drug interaction events using biomedical text

**DOI:** 10.1093/bioadv/vbag243

**Published:** 2026-08-19

**Authors:** Shaoxi Gao, Zhanpeng Gan, Fangfang Han, Yongming Cai

**Affiliations:** College of Medical Information and Engineering, Guangdong Pharmaceutical University, Guangzhou, China; College of Medical Information and Engineering, Guangdong Pharmaceutical University, Guangzhou, China; College of Medical Information and Engineering, Guangdong Pharmaceutical University, Guangzhou, China; NMPA Key Laboratory for Technology Research and Evaluation of Pharmacovigilance, Guangzhou, China; College of Medical Information and Engineering, Guangdong Pharmaceutical University, Guangzhou, China; NMPA Key Laboratory for Technology Research and Evaluation of Pharmacovigilance, Guangzhou, China

## Abstract

**Motivation:**

Unexpected drug–drug interaction events (DDIEs) pose substantial clinical risks, yet many remain unannotated due to data scarcity and the rapid emergence of novel drug combinations. Conventional deep learning approaches struggle to generalize to these unseen interaction types and often lack interpretability under severe class imbalance. To address these challenges, we propose ZSCAN-DDIE, an interpretable zero-shot learning framework for DDIE prediction. The model integrates a biomedical pre-trained language model with an attention-based graph convolutional network (AGCN) to encode DDIE textual semantics and drug molecular structures, respectively. A cross-attention network (CAN) is introduced to align molecular substructures with pharmacological semantic components, enabling fine-grained cross-modal reasoning and improving interpretability at the substructure level. To mitigate modality bias and long-tailed distribution effects, we design a bimodal dynamic alignment (BDA) loss that combines hyperspherical embedding regularization with a stage-adaptive loss-switching mechanism. Experimental results under both conventional and generalized zero-shot settings demonstrate that ZSCAN-DDIE consistently outperforms state-of-the-art baselines across multiple evaluation metrics. The proposed framework not only enhances prediction accuracy for unseen DDIE categories but also provides biologically meaningful insights into molecular interaction mechanisms, offering a robust and clinically relevant solution for pharmacovigilance and drug safety assessment

**Availability and implementation:**

The source code and data are available at https://github.com/GSX-0429/ZSCAN-DDIE.

## 1 Introduction

Drug–drug interactions (DDIs) (Vilar *et al.* 2014) refer to the pharmacological, pharmacodynamic, or pharmacokinetic effects that occur when two or more drugs are administered concomitantly. According to their clinical manifestations, DDIs can be categorized into distinct drug–drug interaction events (DDIEs) (Ryu *et al.* 2018), each representing a specific pharmacological outcome. In DrugBank, a DDIE is defined as “an identifiable event associated with a specific pharmacological effect” (Wishart *et al.* 2018). Unlike conventional DDI prediction, which determines whether an interaction exists, DDIE prediction aims to identify the specific interaction type, providing more clinically informative guidance.

With the rapid approval of new drugs and the increasing complexity of combination therapies, novel drug pairs are continuously introduced into clinical practice. Consequently, previously unobserved DDIEs emerge more frequently. Predicting these unseen interaction events is critical for pharmacovigilance (Wang *et al.* 2024). However, many emerging DDIEs lack sufficient annotations, limiting supervised learning methods. To address this limitation, zero-shot learning (ZSL) has been introduced into DDIE prediction. In the zero-shot DDIE (ZS-DDIE) setting, the model must recognize interaction types that are absent from the training set by leveraging transferable semantic knowledge. Unseen DDIEs usually comprise known semantic attributes in new combinations. For instance, when an unseen DDIE describes that “the serum concentration of Drug1 decreases when combined with Drug2,” the model must associate previously learned semantic components such as “serum concentration” and “decrease” to infer the new event category. This capability requires robust cross-category semantic generalization.

**Figure 1:**
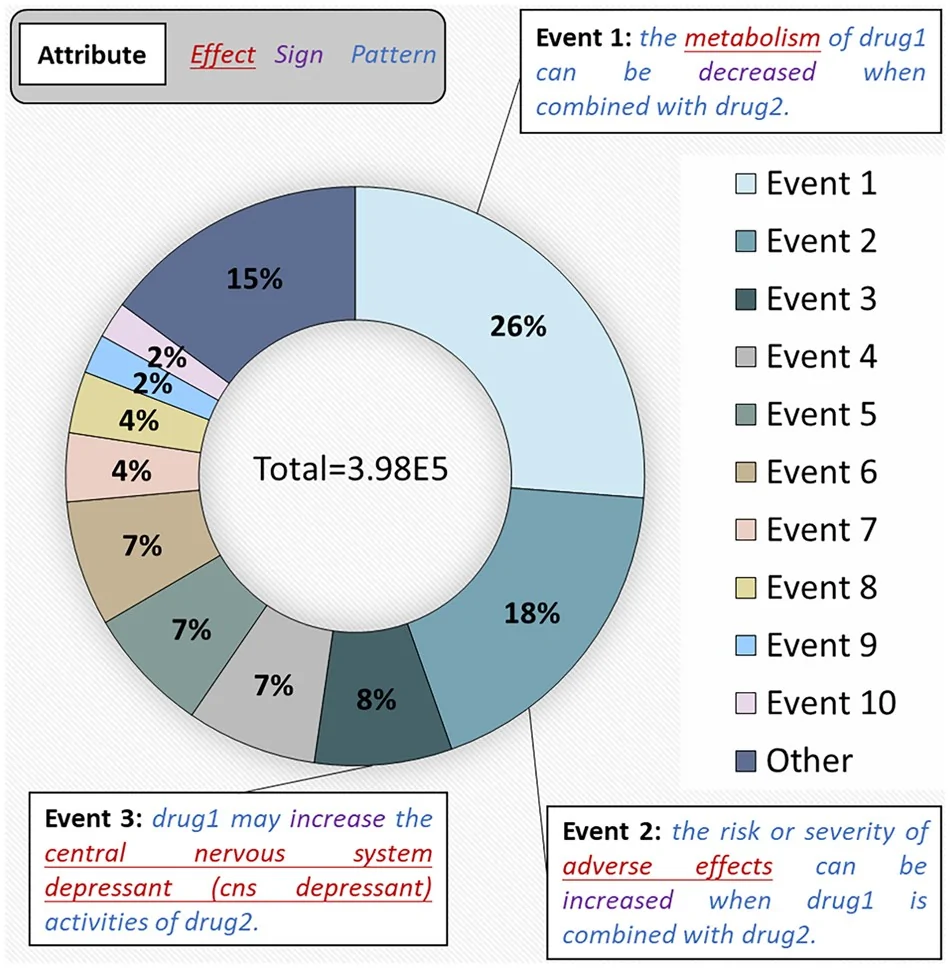
Distribution of DDIE types in the dataset. Nearly all DDIEs can be categorized into three classes: *Effect*, *Sign*, and *Pattern*.

Despite recent advances in deep learning, ZS-DDIE prediction remains challenging for three main reasons. First, annotated samples for rare and emerging DDIEs are extremely limited, making it difficult for supervised models to generalize to unseen categories. Second, most existing methods provide limited interpretability, although DDIEs are inherently associated with molecular-level interactions between functional groups and chemical substructures (Zhang *et al.* 2024). This lack of structural evidence reduces model transparency and limits clinical trust. Third, DDIE datasets exhibit severe class imbalance (Deng *et al.* 2022), where a few frequent categories dominate the data while many interaction types contain only a small number of samples (Fig. 1). Such imbalance distorts decision boundaries and substantially degrades the recognition of minority and unseen classes (Ji *et al.* 2022).

To overcome these limitations, we propose ZSCAN-DDIE, an interpretable zero-shot learning framework that jointly models molecular structures and biomedical semantics for DDIE prediction. Following the compatibility-based ZSL paradigm (Xian *et al.* 2019), drug-pair representations and DDIE semantic representations are projected into a shared embedding space to enable knowledge transfer from seen to unseen categories. BioBERT (Lee *et al.* 2020) is employed to encode biomedical semantic information, while an Attention-based Graph Convolutional Network (AGCN) captures both global molecular topology and pharmacologically meaningful substructures. Furthermore, a Cross-Attention Network (CAN) explicitly aligns molecular substructures with DDIE semantic components, improving cross-modal representation learning and model interpretability. To alleviate modality bias and the long-tailed distribution of DDIE categories, we further introduce a Bimodal Dynamic Alignment (BDA) loss that combines hyperspherical embedding regularization with a stage-adaptive loss-switching strategy (Lin *et al*. 2017), leading to more robust optimization and improved recognition of minority classes.

The main contributions of this work are summarized as follows:

- We propose an Attention-based Graph Convolutional Network (AGCN) that combines gated attention with residual graph convolution to capture both global molecular topology and pharmacologically meaningful substructures for DDIE prediction.
- We develop a Cross-Attention Network (CAN) to establish interpretable alignments between molecular substructures and DDIE semantic components, thereby improving cross-modal representation learning and prediction performance.
- We introduce a Bimodal Dynamic Alignment (BDA) loss that integrates hyperspherical distribution regularization with a stage-adaptive loss-switching strategy to reduce modality bias and improve prediction performance on minority DDIE categories.

Related work is briefly reviewed below, while a more comprehensive survey is provided in the Supplementary Material, available at supplementary material  *Bioinformatics Advances* online.

## 2 Related work

Zero-shot learning (ZSL) has been extensively studied in computer vision and has recently been introduced into biomedical applications to address data scarcity. Existing ZSL methods generally learn a shared embedding space between samples and semantic descriptions, enabling knowledge transfer from seen to unseen classes through compatibility learning (Xian *et al.* 2019). Representative methods employ ranking-based objectives, probabilistic compatibility learning, or contrastive semantic alignment to improve cross-class generalization. Although these methods demonstrate strong transfer capability, they are primarily designed for visual recognition and do not explicitly model molecular structures or biomedical semantic relationships required for DDIE prediction.

Existing DDIE prediction methods can be broadly categorized into DNN-based, GNN-based, and few-shot learning approaches. Early studies employed multimodal deep neural networks to integrate molecular fingerprints, targets, enzymes, and pathways for interaction event prediction. More recent GNN-based models exploit molecular graphs and drug interaction networks to capture structural dependencies, while few-shot learning methods alleviate data scarcity for rare interaction events. Nevertheless, these methods still require labeled samples for each interaction type and therefore cannot generalize to completely unseen DDIE categories.

Recently, several studies have begun to investigate zero-shot DDIE prediction. 3DGT-DDI (Lv *et al.* 2023) introduces semantic representations derived from biomedical texts to support cold-start prediction, while ZeroDDI (Wang *et al.* 2024) further improves semantic representation learning through biomedical language models and dual-modal alignment. However, these methods still suffer from three important limitations. First, they mainly provide prediction results without revealing interpretable molecular mechanisms. Second, cross-modal alignment is performed at the global representation level, ignoring fine-grained correspondence between molecular substructures and semantic components. Third, severe class imbalance remains insufficiently addressed, resulting in degraded performance for rare DDIE categories.

## 3 Materials and methods

### 3.1 Datasets

We evaluate on the ZS-DDIE benchmark (Wang *et al.* 2024), constructed from DrugBank v5.1.9 (Wishart *et al.* 2018) and the MeSH database. The dataset comprises 2,004 approved small-molecule drugs, 3,94,118 DDIs, and textual descriptions for 175 distinct DDIE types, with drug names normalized using standardized placeholders “Drug1” and “Drug2.” Following (Deng *et al.* 2020, Feng *et al.* 2023), each DDIE text is decomposed into Effect, Sign, and Pattern attributes. StanfordNLP (Qi *et al.* 2018) extracts Effect keywords mapped to MeSH (yielding 114 attributes), Sign indicates directionality (increase/decrease), and Pattern covers three basic syntactic structures. To simulate zero-shot learning, the 175 DDIE types are partitioned by frequency into 107 high-frequency seen classes and 68 low-frequency unseen classes. Detailed hyperparameters and experimental settings are provided in Table S1, available at supplementary material  *Bioinformatics Advances* online. All experiments are independently repeated five times, reporting the mean performance with standard deviation.

### 3.2 The framework of ZSCAN-DDIE method

**Figure 2:**
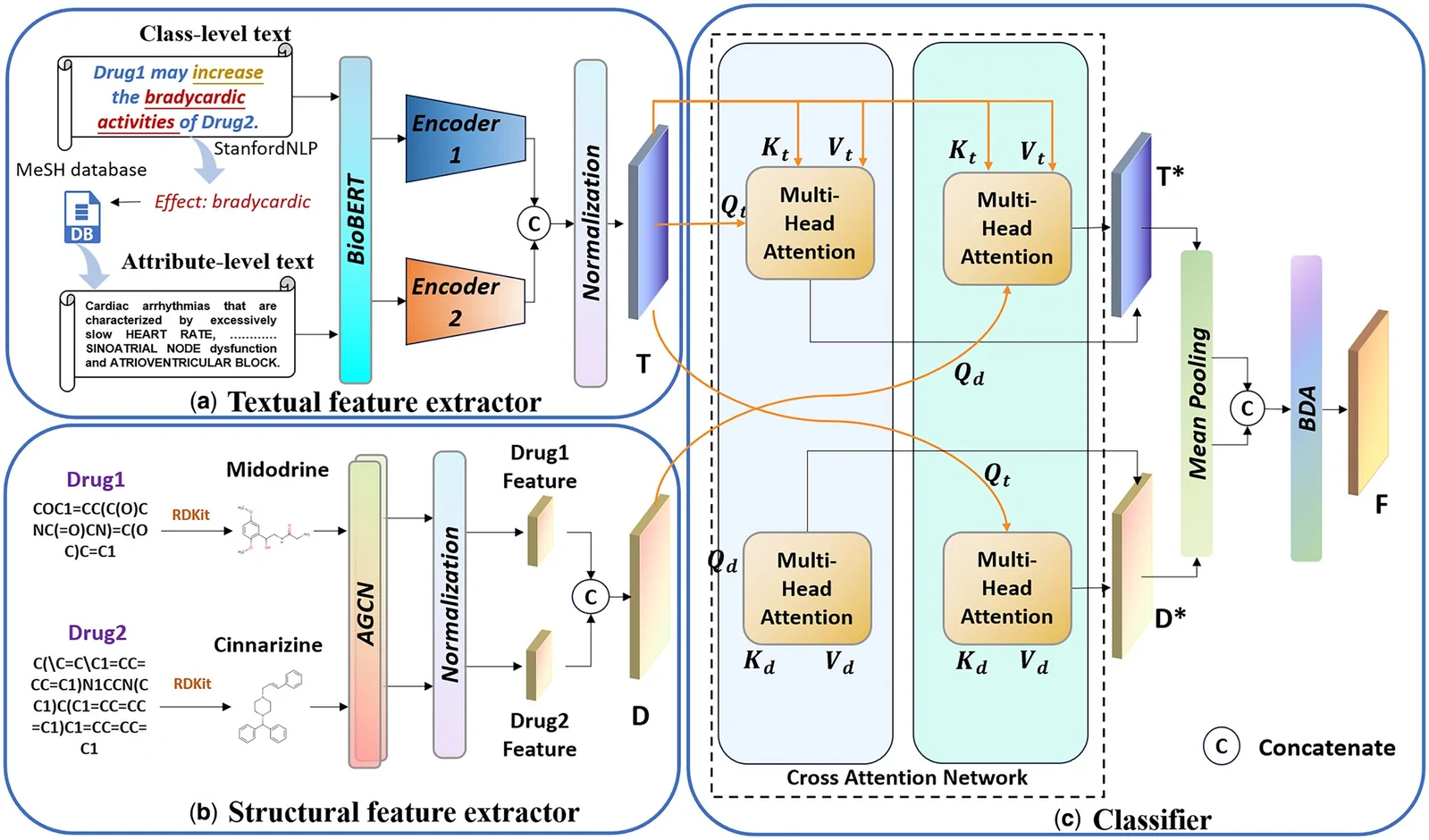
The overall framework of ZSCAN-DDIE. The model consists of two parallel feature extraction encoders that generate semantic representations *T* and drug pairs representations *D*, respectively. *CAN*: It fuses *T* and *D* through multi-head, self-attention and cross-attention. Then fused representations T* and D* are concatenated into *F* after mean pooling.

The technical framework of our ZSCAN-DDIE is illustrated in Fig. 2. It consists of three main components: a text feature extractor, a structural feature extractor, and a classifier.

#### 3.2.1 The LLM-based textual feature extractor

In the ZS-DDIE prediction task, unseen classes of DDIEs lack annotated data, making it necessary to rely on semantic transfer mechanisms for inference. Given that DDIE-related texts often lack explicit semantic representations of biological characteristics, we draw inspiration from (Wang *et al.* 2024) and propose a dual-channel semantic embedding framework for feature fusion, as illustrated in Fig. 2a. Specifically, we employ BioBERT, which has been pretrained on large-scale biomedical corpora, to encode both class-level descriptions (e.g. “enhanced bradycardia”) and MeSH-based attribute definitions (e.g. the definition of “bradycardia”). These two semantic types are projected into a shared embedding space, and effect-specific attributes are incorporated to enhance biological interpretability.

To achieve this, we extract semantic representations from the class-level text and the attribute-level text introduced in Dataset section. BioBERT is applied separately to encode each type of text: for class-level text, we obtain a token feature set ${\left\{h_{m}^{\mathrm{cls}}\in R^{{\mathrm{d}}_{t}}\right\}}_{m=1}^{M}$​, and for attribute-level text, we obtain ${\left\{e_{l}^{\mathrm{attr}}\in R^{{\mathrm{d}}_{t}}\right\}}_{l=1}^{L}$​, where M and L denote the number of tokens, and $d_{t}$​ is the original feature dimension. To ensure dimensional consistency, both sets of features are projected from $d_{t}$​ to a lower-dimensional space ${\mathrm{d}}_{r}$ using separate Multilayer Perceptron (MLP).


(1) $$H^{\mathrm{cls}}=[{\mathrm{MLP}}_{\mathrm{cls}}(h_{1}^{\mathrm{cls}}),\ldots ,{\mathrm{MLP}}_{\mathrm{cls}}(h_{M}^{\mathrm{cls}})]$$

(2) $$E^{\mathrm{attr}}=\frac{1}{L}\sum_{l=1}^{L}{\mathrm{MLP}}_{\mathrm{attr}}(e_{l}^{\mathrm{attr}})$$

We then broadcast the attribute-level feature vector to match the shape of the class-level sequence and construct a two-level semantic representation T by concatenation and layer normalization:


(3) $$T=LN(\mathrm{Concat}(H^{\mathrm{cls}},E^{\mathrm{attr}}⊗1_{M}))\in R^{{(M+1)\times \mathrm{d}}_{t}}$$

where LN denotes layer normalization, ⊗ represents feature broadcasting, and $1_{M}$ is a vector of ones. This design injects global attribute semantics into the local context of class features, enhancing biological expressiveness and enabling the model to transfer pharmacological patterns from seen to unseen classes. It thus supports effective zero-shot prediction based on semantic generalization.

Together, this dual-channel semantic embedding framework enhances the model’s ability to semantically align unnoticed DDIEs with known pharmacological patterns, fulfilling the zero-shot learning goal of transferring knowledge beyond labeled categories.

#### 3.2.2 The AGCN-based structural feature extractor

Drug molecular graphs provide critical topological and chemical information for characterizing interaction patterns (Lu *et al.* 2024). However, conventional GNNs face two main limitations: they struggle to simultaneously capture local bond features and global topology, and they lack dynamic perception of crucial substructures. To address this, we propose an Attention-based Graph Convolutional Network (AGCN), which combines gated-attention feature fusion with residual-connected convolution operations (Kang *et al.* 2022) to enable collaborative multi-level feature modeling.

**Figure 3:**
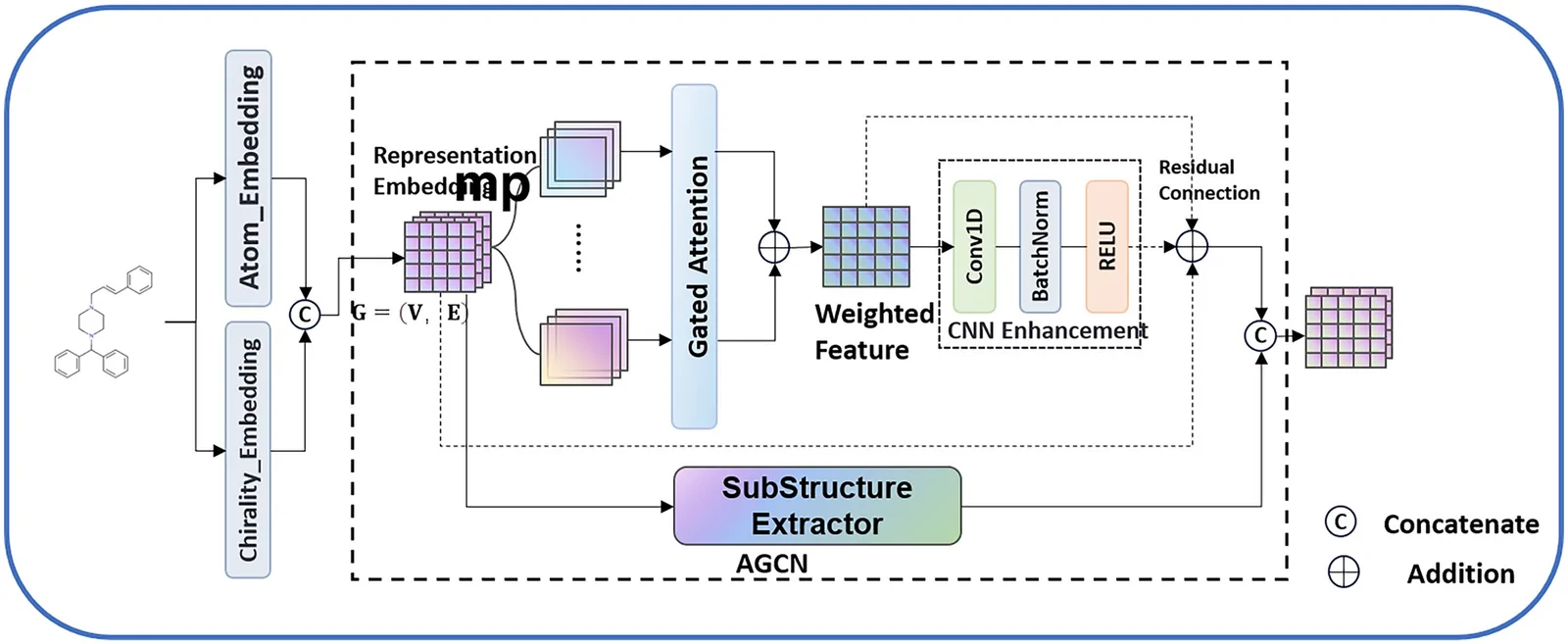
Framework of the AGCN.

As illustrated in Fig. 3, AGCN comprises a hybrid convolutional layer and a substructure extraction module. The hybrid layer integrates learnable attention coefficients, CNN-based local spatial enhancements, and residual connections to balance global topology with local atomic semantics while mitigating overfitting. Meanwhile, the substructure extractor leverages self-attention to identify pharmacologically active functional groups and construct cross-drug substructure similarity matrices, providing explicit structural evidence to enable zero-shot knowledge transfer to novel DDIE classes.

We begin by parsing each drug molecule into a graph structure G=(V,E) using the RDKit toolkit, where V denotes the set of nodes representing atoms and E⊂V×V denotes the set of edges corresponding to chemical bonds. Based on this topological representation, we construct the AGCN architecture, which captures both global molecular representations and key substructure features through a multi-level feature extraction mechanism.

The hybrid convolutional layer in AGCN integrates three complementary feature processing components. First, diverging from traditional Graph Attention Network (GAT) that employ a fixed number of attention heads, we design an adaptive gated-attention for weight computation. For a center node i and its neighbor j, we apply independent linear transformations $W_{\mathrm{node}}$ and $W_{\mathrm{neight}}$​ to project their original features into an interaction space. The resulting vectors are concatenated and passed through a single-layer perceptron to compute the attention coefficient:


(4) $$\alpha_{ij}=\sigma (W_{a}^{T}[W_{\mathrm{node}}x_{i}∥W_{\mathrm{neight}}x_{j}]+b_{a})$$

where $W_{a}$ and $b_{a}$ are learnable parameters, and ∥ denotes vector concatenation. This design not only captures pairwise interaction strength between nodes but also ensures interpretability through the sigmoid activation σ; a higher value of $\alpha_{ij}$ indicates a stronger contribution of the corresponding chemical bond to the interaction. After aggregating the neighbor features weighted by $\alpha_{ij}$, the CNN Enhancement module applies zero-padding and a 1D convolution with a kernel size of 3 to extract local spatial patterns. The resulting feature maps are passed through Batch Normalization and ReLU activation to produce enhanced representations:


(5) $$h_{i}^{\mathrm{cnn}}=\mathrm{ReLU}(BN({\mathrm{Conv}1D}_{k=3}(\sum_{j\in N(i)}\alpha_{ij}x_{j})))$$

Finally, the residual connection integrates the original node features with the attention-aggregated features and the CNN-enhanced features using learnable scaling factors $\beta_{1}$ and $\beta_{2}$:


(6) $$h_{i}^{\mathrm{out}}=x_{i}+\beta_{1}\cdot h_{i}^{\mathrm{cnn}}+\beta_{2}\cdot \sum_{j\in N(i)}\alpha_{ij}x_{j}$$

To systematically capture functional units mediated by specific molecular substructures in drug–drug interactions, AGCN further incorporates a substructure extractor. This module leverages a self-attention mechanism to assess the importance of different substructures within the molecular graph and generate their corresponding embedding representations. The process consists of three stages:

First, a set of learnable query vectors $Q\in R^{K\times d}$(where K is the predefined number of substructures) is used to compute attention distributions over the node features:


(7) $$A=\mathrm{softmax}(\frac{QW_{Q}^{T}{\left(KW_{K}^{T}\right)}^{T}}{\sqrt{d}})$$

where $W_{Q}$ and $W_{K}$ are projection matrices, and $A\in R^{N\times K}$ denotes the probability distribution indicating the degree to which each node is associated with each substructure.

Second, the node features are aggregated according to the attention weights to form K substructure embeddings:


(8) $$S_{k}=\sum_{i=1}^{N}A_{ki}\cdot (Vx_{i})$$

where V is the value projection matrix, and each $S_{k}$ corresponds to a latent pharmacophore or functional group.

Finally, for a given drug pair $\left(d_{1},d_{2}\right)$, we obtain two sets of substructure embeddings, ${\left\{S_{k}^{d_{1}}\right\}}_{K=1}^{K}$ and ${\left\{S_{k}^{d_{2}}\right\}}_{K=1}^{K}$, and compute the cross-drug substructure association matrix using cosine similarity:


(9) $$M_{ij}=\frac{S_{i}^{d_{1}}\cdot S_{j}^{d_{2}}}{\left\|S_{i}^{d_{1}}\|\|S_{j}^{d_{2}}\right\|}$$

The resulting matrix $M_{ij}$ provides an interpretable visualization of potential interacting substructures across the two drugs. This explicit quantification of cross-drug substructure relationships offers biologically meaningful insights and serves as a functional basis for zero-shot DDIE prediction.

In summary, AGCN plays an indispensable role in the ZSCAN-DDIE framework by capturing both local and global structural patterns, identifying functionally crucial molecular substructures, and enabling biologically interpretable predictions under the zero-shot setting where no annotated samples for novel interactions are available.

#### 3.2.3 Cross-attention-network-based feature fusion module

The lack of interpretability in DDIE prediction undermines clinical adoption, as traditional feature fusion methods often ignore fine-grained associations between drug molecular substructures and biomedical text descriptions. To address this limitation in zero-shot scenarios, inspired by ProST (Xu *et al.* 2023), we propose a Cross-Attention Network (CAN) to dynamically model cross-modal dependencies. CAN employs drug molecular representations as Queries and semantic embeddings as Key/Value inputs within a multi-head attention mechanism. By computing cross-modal similarity and aligning local molecular patterns with global semantic descriptions in a shared latent space, CAN produces a context-aware joint representation, significantly boosting both model interpretability and zero-shot generalization to unseen DDIE classes

To further enable cross-modal alignment and enhance interpretability, we design a joint representation generation mechanism, which integrates molecular and semantic features into a discriminative cross-domain interaction representation. The fused representation F is constructed by concatenating pooled features from drug molecules $D\in R^{m\times d_{p}}$ and semantic embeddings $\in R^{{(M+1)\times \mathrm{d}}_{t}}$:


(10) F=Concat(MeanPool(D,dim=1),MeanPool(T,dim=1))

Here, MeanPool computes the element-wise average across the sequence dimension, and Concat denotes the concatenation operation. The concatenated features are then refined via both multi-head self-attention and cross-attention mechanisms:


(11) $$D=\frac{1}{2}[\mathrm{MHA}(Q_{p},K_{p},V_{p})+\mathrm{MHA}(Q_{t},K_{p},V_{p})]$$

(12) $$T=\frac{1}{2}[\mathrm{MHA}(Q_{t},K_{t},V_{t})+\mathrm{MHA}(Q_{p},K_{t},V_{t})]$$

Here, MHA denotes multi-head attention, and the output dimensions match those of the inputs. $Q_{p},K_{p},V_{p}\in R^{m\times d_{p}}$ and $Q_{t},K_{t},V_{t}\in R^{{(M+1)\times \mathrm{d}}_{t}}$ are the queries, keys, and values derived from drug and semantic features, respectively. These are projected via the following linear transformations:


(13) $$DQ_{p}=DW_{q}^{p},\;{\;K}_{p}=DW_{k}^{p},\;{\;V}_{p}=DW_{v}^{p}$$

(14) $$Q_{t}=TW_{q}^{t},\;{\;K}_{t}=TW_{k}^{t},\;{\;V}_{t}=TW_{v}^{t}$$

where $W_{q}^{p},\;W_{k}^{p},\;W_{v}^{p}\in R^{{d_{p}\times \mathrm{d}}_{p}}$ and $W_{q}^{t},\;W_{k}^{t},\;W_{v}^{t}\in R^{{d_{t}\times \mathrm{d}}_{t}}$ are the projection matrices for drug and semantic features, respectively. This bidirectional projection ensures that molecular and DDIE semantic embeddings are aligned within a unit hypersphere, thus mitigating modality bias in zero-shot scenarios.

By jointly leveraging self-attention and cross-attention mechanisms, this fusion module precisely captures dependencies between drug molecules and DDIE semantics. Beyond boosting prediction accuracy, the attention maps generated during this process offer interpretable evidence by identifying which molecular substructures most contribute to a specific pharmacological effect. This capability enables visual validation of model reasoning and supports mechanism-level understanding of drug interactions.

By bridging molecular-level evidence with semantic-level meaning, this module strengthens both the transparency and clinical relevance of the final prediction—key advantages in safety-critical drug interaction scenarios.

#### 3.2.4 Bimodal dynamic alignment loss module

**Figure 4:**
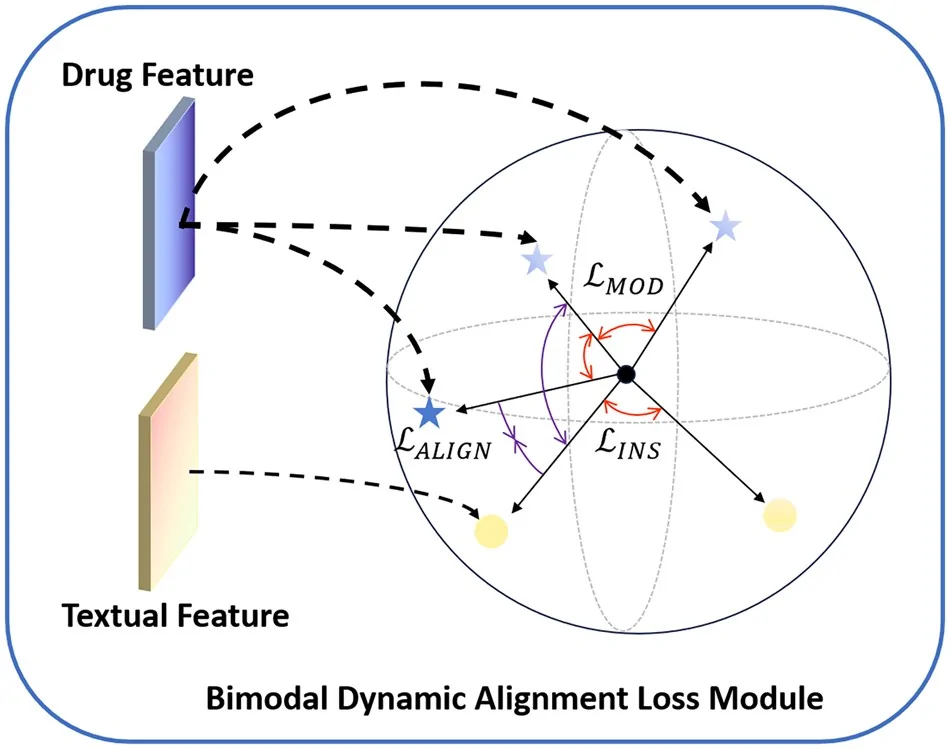
Bimodal dynamic alignment loss module.

The ZS-DDIE prediction task presents two core challenges: (i) a modality gap between molecular representations of drug pairs and the semantic representations of DDIEs, which may lead to semantic misalignment when fused directly; and (ii) a long-tailed distribution of DDIE classes, where the scarcity of minority class samples severely undermines the model’s discriminative capability. To address these issues, inspired by (Wang *et al.* 2024), we propose a Bimodal Dynamic Alignment (BDA) loss module, as shown in Fig. 4, which facilitates cross-modal alignment and imbalance-aware optimization through the following components:

1. Cross-Modal Contrastive Alignment Loss ($L_{\mathrm{ALIGN}}$): Encourages alignment between the molecular representation of a drug pair and the embedding of its ground-truth DDIE semantics by minimizing their distance in the shared embedding space.
2. Bimodal Spherical Distribution Loss ($L_{\mathrm{BSD}}$): Enforces uniform distribution of all representations (molecular and semantic) on a unit hypersphere to prevent representation collapse and enhance inter-class separability.
3. Stage-Adaptive Classification Loss ($L_{\mathrm{CLS}}$): Dynamically switches between cross-entropy loss and focal loss during training phases to balance stability and the recognition of rare classes.

The overall loss function is defined as:


(15) $$L_{\mathrm{Total}}={\lambda_{1}L}_{\mathrm{ALIGN}}+\lambda_{2}L_{\mathrm{BSD}}+L_{\mathrm{CLS}}$$

where $\lambda_{1}$ and $\lambda_{2}$ are hyperparameters that control the contributions of the alignment and distribution components, respectively. Following the loss weighting strategy adopted in ZeroDDI (Wang *et al.* 2024), $\lambda_{1}$ and $\lambda_{2}$ were set to 1 and 0.7, respectively. This setting maintains the alignment loss as the primary optimization objective while introducing the distribution regularization term with an appropriate contribution to improve the discriminative ability of learned representations.

*Cross-Modal Contrastive Alignment Loss*. To bridge the representation gap between heterogeneous molecular graph features and DDIE textual features, we design a Cross-Modal Contrastive Alignment Loss ($L_{\mathrm{ALIGN}}$) inspired by contrastive learning (Han *et al.* 2022). It pulls positive drug-text pairs closer while pushing negative pairs apart in the shared space to enforce cross-modal semantic consistency.

Let $D_{i}$ denote the molecular representation of the i-th drug pair, and $T_{j}$ denote the semantic embedding of the j-th DDIE class. The contrastive alignment loss is formulated using normalized cosine similarity as follows:


(16) $$L_{\mathrm{ALIGN}}=\sum_{i=1}^{N}\log\left[\frac{\;\text{exp}\;(\kappa \cdot \frac{D_{i}^{⊤}T_{y_{i}}}{\left\|D_{i}\|\|T_{y_{i}}\right\|})}{\sum_{j=1}^{C}\exp(\kappa \cdot \frac{D_{i}^{⊤}T_{j}}{\left\|D_{i}\|\|T_{j}\right\|})}\right]$$

Here, $T_{y_{i}}$​​ is the ground-truth semantic embedding for the DDIE class corresponding to drug pair i, C is the total number of DDIE classes, and κ is a learnable temperature coefficient that adaptively scales cross-modal similarity during training via backpropagation.

*Bimodal Spherical Distribution Loss*. Existing multimodal methods often overlook geometric embedding distributions, causing representation collapse or class overlap. Inspired by hyperspherical manifold learning (Lu *et al.* 2022), we propose a Bimodal Spherical Distribution Loss ($L_{\mathrm{BSD}}$) to constrain representations onto a unit hypersphere, maximizing inter-class separability while balancing both modalities. It comprises two components:

1. Intra-Modal Uniformity Loss ($L_{\mathrm{MOD}}$): This term encourages DDIE semantic embeddings $T_{i}^{j}$ to be evenly distributed on the unit hypersphere centered around the class-wise global mean $\mu_{i}=\frac{1}{\left|C\right|}\sum_{j}T_{i}^{j}$, ensuring semantic disentanglement:
  (17) LMOD=1N|C|∑i=1N∑j=1|C|{1+MAXk∈C[(Tij-μj)‖Tij-μj‖·(Tik-μj)‖Tik-μj‖]}
2. Instance Contrast Loss ($L_{\mathrm{INS}}$): This component constrains each drug-pair representation $D_{i}$ to remain close to its corresponding class center $\mu_{j}$, while being distant from centers of other classes:
  (18) LINS=1N∑i=1N{1+MAXk∈S[(Di-μj)‖Di-μj‖·(Dk-μj)‖Dk-μj‖]}

where S denotes the set of all drug-pair samples. The final Bimodal Spherical Distribution Loss is given by:


(19) $$L_{\mathrm{BSD}}=L_{\mathrm{MOD}}+L_{\mathrm{INS}}$$

This dual-constraint formulation ensures balanced dispersion of semantic and molecular representations across the hyperspherical embedding space, thus enhancing robustness and class separability in the zero-shot DDIE prediction setting.

*Stage-Adaptive Classification Loss*. To tackle severe DDIE class imbalance without causing early-stage training instability, we propose a Stage-Adaptive Classification Loss ($L_{\mathrm{CLS}}$). It uses standard cross-entropy in early training for stability and transitions to a dynamic focal loss in later epochs for enhanced optimization on rare classes.

Let T be the total number of training epochs and t the current epoch. The classification loss $L_{\mathrm{CLS}}$​ is defined as a stage-dependent function that dynamically transitions between the two loss forms:


(20) LCLS={-∑i=1Nωyilog⁡p(yi|uDi)            t≤T3-∑i=1Nαt(1-p(yi|Di))βlog⁡p(yi|Di) t>T3

where β is the focusing parameter, $\omega_{y_{i}}$ denotes the prior weight of class $y_{i}$, and $\alpha_{t}=\frac{t}{T}$​ is a temporal decay factor that progressively increases the relative importance of hard-to-classify samples. This design enables the model to enhance gradient feedback for tail classes during the later training stages while ensuring a smooth transition through $\alpha_{t}$, thereby avoiding abrupt shifts in the optimization direction.

This loss module is specifically designed to ensure ZSCAN-DDIE remains robust under modality bias and class imbalance-two defining challenges of the ZS-DDIE prediction task—while enhancing discrimination across rare events.

## 4 Results and discussion

### 4.1 Evaluation metrics

We evaluate under Conventional Zero-Shot Learning (CZSL) and Generalized Zero-Shot Learning (GZSL). CZSL uses only unseen classes in test set; metrics include average accuracy (${\mathrm{ACC}}_{\mathrm{ave}}^{u}$), top-k accuracy (${\mathrm{ACC}}_{@k}^{u}$), F1, AUPR, AUC. GZSL includes both seen and unseen; metrics include ${\mathrm{ACC}}_{\mathrm{ave}}^{s}$, ${\mathrm{ACC}}_{\mathrm{ave}}^{u}$, harmonic mean (${\mathrm{ACC}}^{H}$) (Xian *et al.* 2019), F1, AUPR, AUC.

### 4.2 Baselines

We compared ZSCAN-DDIE against state-of-the-art DDIE prediction models and standard zero-shot learning (ZSL) baselines. All baselines share the same drug structural feature extractor as our framework. Standard ZSL baselines evaluate three compatibility loss functions—ZSLCE (Sumbul *et al.* 2013; cross-entropy loss), ZSLHinge (Frome et al. 2018; hinge ranking loss), and ZSLTriplet (Cacheux *et al.* 2019; triplet loss with a tunable semantic margin)—combined with both class-based (PLM embeddings) and attribute-based (binary vectors) DDIE semantic representations. Additionally, we included two competitive DDIE-specific models: 3DGT-DDI (Lv *et al.* 2023), a 3D GNN and few-shot learning model using a PLM-CNN semantic encoder; and ZeroDDI (Wang *et al.* 2024), a zero-shot model featuring biomedical semantics-enhanced representation learning and dual-modality alignment.

### 4.3 Comparison with baselines

Tables 1 and 2 report the mean performance and standard deviations over five independent runs under CZSL and GZSL scenarios. Across both scenarios, ZSCAN-DDIE consistently outperforms existing baselines with low standard deviations, demonstrating superior performance, stability, and repeatability.

**Table 1:** Mean performance (± standard deviation) over five independent runs for ZSCAN-DDIE and baseline methods under the CZSL scenarios.

Method	CZSL						
	${\mathrm{ACC}}_{\mathrm{ave}}^{u}$	${\mathrm{ACC}}_{@1}^{u}$	${\mathrm{ACC}}_{@3}^{u}$	${\mathrm{ACC}}_{@5}^{u}$	F1	AUPR	AUC
ZSLCE	0.081 ± 0.001	0.166 ± 0.002	0.387 ± 0.003	0.604 ± 0.004	0.225 ± 0.005	0.417 ± 0.001	0.793 ± 0.002
ZSLHinge	0.128 ± 0.004	0.107 ± 0.005	0.287 ± 0.001	0.425 ± 0.002	0.213 ± 0.003	0.438 ± 0.004	0.779 ± 0.005
ZSLTriplet	0.191 ± 0.002	0.264 ± 0.003	0.514 ± 0.004	0.651 ± 0.005	0.232 ± 0.001	0.499 ± 0.002	0.829 ± 0.003
3DGT-DDI	0.118 ± 0.005	0.256 ± 0.001	0.522 ± 0.002	0.624 ± 0.003	0.223 ± 0.004	0.530 ± 0.005	0.832 ± 0.001
ZeroDDI	0.151 ± 0.003	0.208 ± 0.004	0.484 ± 0.005	0.651 ± 0.001	0.276 ± 0.002	0.512 ± 0.003	0.850 ± 0.004
ZSCAN-DDIE	**0.230 ± 0.001**	**0.299 ± 0.001**	**0.569 ± 0.002**	**0.722 ± 0.002**	**0.293 ± 0.002**	**0.602 ± 0.002**	**0.883 ± 0.002**

The best and suboptimal results are highlighted in **bold** and underline, respectively.

**Table 2:** Mean performance (± standard deviation) over five independent runs for ZSCAN-DDIE and baseline methods under the GZSL scenarios.

Method	GZSL					
	${\mathrm{ACC}}_{\mathrm{ave}}^{s}$	${\mathrm{ACC}}_{\mathrm{ave}}^{u}$	${\mathrm{ACC}}^{H}$	F1	AUPR	AUC
ZSLCE	0.588 ± 0.003	0.080 ± 0.004	0.140 ± 0.005	0.286 ± 0.001	0.440 ± 0.002	0.841 ± 0.003
ZSLHinge	0.666 ± 0.001	0.115 ± 0.002	0.196 ± 0.003	0.303 ± 0.004	0.466 ± 0.005	0.840 ± 0.001
ZSLTriplet	0.739 ± 0.004	0.162 ± 0.005	0.266 ± 0.001	0.300 ± 0.002	0.512 ± 0.003	0.870 ± 0.004
3DGT-DDI	0.777 ± 0.002	0.119 ± 0.003	0.206 ± 0.004	0.338 ± 0.005	0.564 ± 0.001	0.867 ± 0.002
ZeroDDI	0.755 ± 0.005	0.123 ± 0.001	0.212 ± 0.002	**0.343 ± 0.003**	0.539 ± 0.004	0.890 ± 0.005
ZSCAN-DDIE	**0.783 ± 0.002**	**0.222 ± 0.003**	**0.345 ± 0.003**	0.322 ± 0.004	**0.588 ± 0.004**	**0.905 ± 0.004**

The best and suboptimal results are highlighted in **bold** and underline, respectively.

In the CZSL scenario, ZSCAN-DDIE achieves top performance across all metrics on unseen categories. Traditional ZSL methods (e.g. ZSLHinge and ZSLTriplet) perform significantly worse due to their lack of domain adaptation, validating the necessity of cross-modal semantic alignment for zero-shot DDIE prediction.

In the more practically relevant GZSL scenario, ZSCAN-DDIE further demonstrated its balanced discriminative capability across both seen and unseen classes. It achieved a substantially higher average accuracy on unseen classes compared to other models, and its harmonic mean accuracy exceeded that of the second-best model by more than 40%. This highlighted its superiority in cross-class generalization and adaptation to long-tailed distributions. Furthermore, traditional zero-shot learning methods (e.g. ZSLHinge and ZSLTriplet), due to the absence of cross-modal alignment mechanisms, showed significant performance gaps in robustness-related metrics such as AUPR and AUC when compared to ZSCAN-DDIE.

Furthermore, the GZSL setting inherently evaluates the model’s performance on “common” DDIEs (i.e. the seen classes with sufficient labeled data). As shown in Table 2, our model achieves a high accuracy of 0.783 on seen classes (${\mathrm{ACC}}_{\mathrm{ave}}^{s}$), outperforming all baseline methods. This result confirms that ZSCAN-DDIE is not only effective in the challenging zero-shot scenario but also a robust and superior model for conventional DDIE prediction tasks where ample training data is available. These findings confirm that ZSCAN-DDIE effectively mitigates modality bias and class imbalance. By integrating AGCN structural modeling, cross-attention-driven feature fusion, and dynamic loss optimization, our framework delivers a robust and generalizable solution for identifying unobserved DDIEs.

Beyond benchmarks, ZSCAN-DDIE shows strong potential for pharmacovigilance knowledge discovery. Relying on semantic transfer rather than label memorization, it infers plausible interactions for unseen categories or new drug pairs, offering prioritized hypotheses for pharmacological validation. Moreover, the attention alignment between molecular substructures and biomedical semantics provides interpretable evidence to facilitate safety assessment.

### 4.4 Comparative experiments on AGCN

To evaluate AGCN in the ZS-DDIE prediction task, we compared it against four mainstream GNN baselines—GCN (Fu *et al.* 2021), GraphSAGE (Hamilton *et al.* 2017), GIN (Xu *et al.* 2018), and GAT (Veličković *et al.* 2017)—under both CZSL and GZSL scenarios (Tables 3 and 4).

**Table 3:** Mean performance (± standard deviation) of AGCN and Mainstream GNNs under the CZSL Scenario.

Method	CZSL						
	${\mathrm{ACC}}_{\mathrm{ave}}^{u}$	${\mathrm{ACC}}_{@1}^{u}$	${\mathrm{ACC}}_{@3}^{u}$	${\mathrm{ACC}}_{@5}^{u}$	F1	AUPR	AUC
GCN	0.109 ± 0.003	0.122 ± 0.004	0.345 ± 0.002	0.523 ± 0.005	0.169 ± 0.001	0.433 ± 0.003	0.820 ± 0.004
GraphSAGE	0.083 ± 0.002	0.136 ± 0.005	0.334 ± 0.003	0.506 ± 0.001	0.170 ± 0.004	0.446 ± 0.002	0.794 ± 0.005
GIN	0.170 ± 0.004	0.264 ± 0.001	0.507 ± 0.005	0.669 ± 0.003	0.231 ± 0.002	0.572 ± 0.004	0.854 ± 0.001
GAT	0.173 ± 0.005	0.280 ± 0.003	0.542 ± 0.002	0.686 ± 0.004	0.266 ± 0.001	0.600 ± 0.005	0.865 ± 0.003
AGCN	**0.230 ± 0.001**	**0.299 ± 0.001**	**0.569 ± 0.002**	**0.722 ± 0.002**	**0.293 ± 0.002**	**0.602 ± 0.002**	**0.883 ± 0.002**

The best and suboptimal results are highlighted in bold and underline, respectively.

**Table 4:** Mean performance (± standard deviation) of AGCN and Mainstream GNNs under the GZSL Scenario.

Method	GZSL					
	${\mathrm{ACC}}_{\mathrm{ave}}^{s}$	${\mathrm{ACC}}_{\mathrm{ave}}^{u}$	${\mathrm{ACC}}^{H}$	F1	AUPR	AUC
GCN	0.631 ± 0.003	0.086 ± 0.004	0.151 ± 0.002	0.287 ± 0.005	0.470 ± 0.001	0.875 ± 0.004
GraphSAGE	0.572 ± 0.002	0.080 ± 0.003	0.140 ± 0.005	0.275 ± 0.001	0.448 ± 0.004	0.852 ± 0.002
GIN	0.693 ± 0.005	0.156 ± 0.001	0.254 ± 0.003	0.300 ± 0.004	0.559 ± 0.002	0.884 ± 0.005
GAT	0.705 ± 0.001	0.189 ± 0.004	0.298 ± 0.002	0.311 ± 0.003	0.571 ± 0.005	0.890 ± 0.001
AGCN	**0.783 ± 0.002**	**0.222 ± 0.003**	**0.345 ± 0.003**	**0.322 ± 0.004**	**0.588 ± 0.004**	**0.9048 ± 0.004**

The best and suboptimal results are highlighted in bold and underline, respectively.

Under the CZSL scenario, AGCN significantly outperforms all baselines across all evaluation metrics on unseen classes. This indicates that combining gated attention with residual convolution enables AGCN to more accurately capture local substructure interaction patterns within drug molecules, thereby enhancing zero-shot reasoning for novel DDIE categories.

Under the GZSL scenario, AGCN demonstrates superior overall performance, achieving a substantially higher harmonic mean while preserving strong discrimination on seen classes and improving unseen class recognition. These findings confirm that hierarchical substructure extraction and cross-drug substructure alignment effectively enhance model generalization and reduce traditional GNNs’ overfitting bias toward seen classes.

### 4.5 Ablation experiments

**Figure 5:**
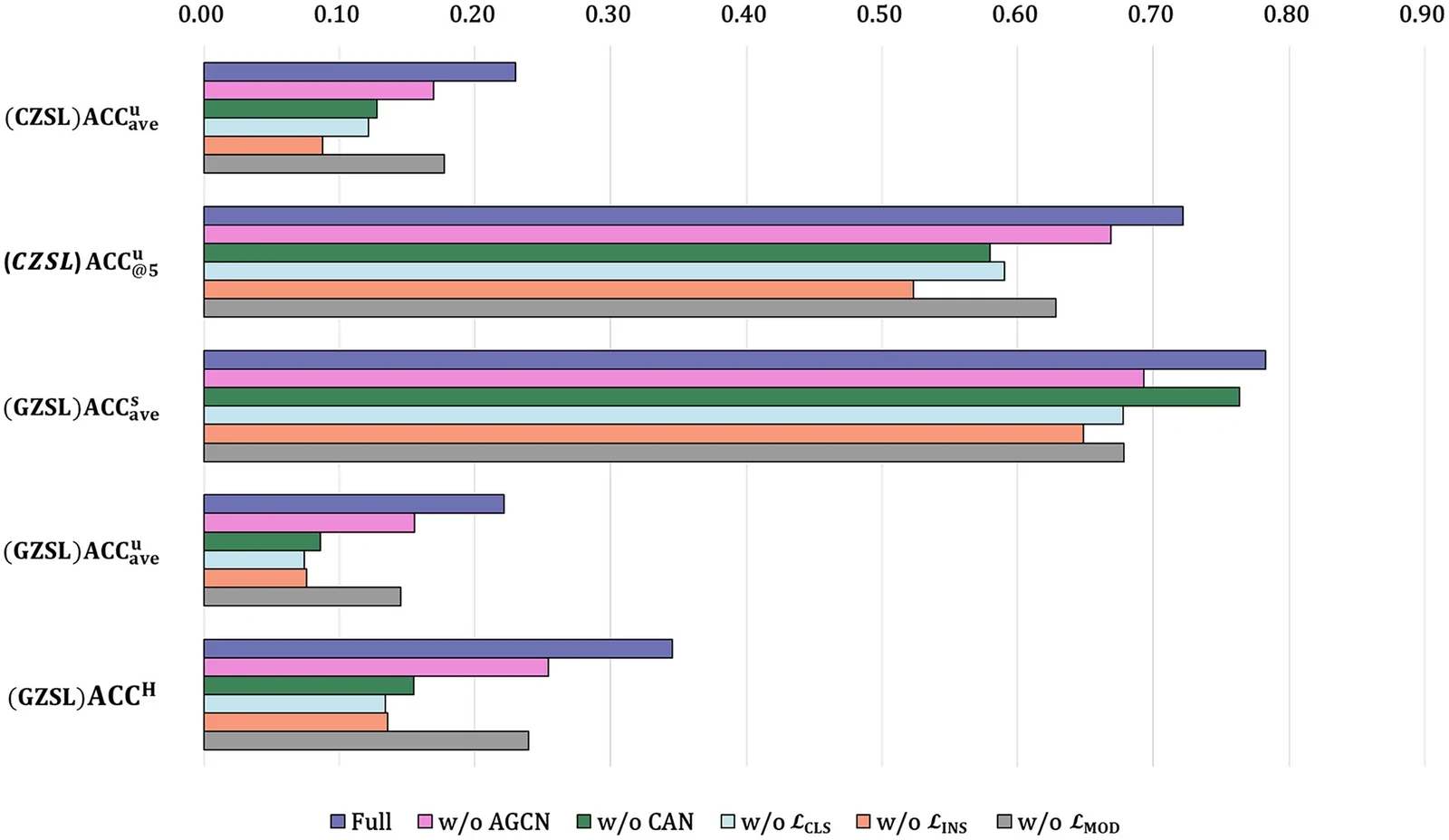
Performance comparison between ZSCAN-DDIE and its ablated variants under the CZSL and GZSL scenarios.

Ablation studies were conducted to quantitatively assess the effectiveness of each component in the ZSCAN-DDIE. We compared the performance of the complete model (Full) with several ablated versions: removing the structural encoder (w/o AGCN), replacing the cross-attention fusion module (w/o CAN), and eliminating components of the bimodal dynamic alignment loss module (${w/o\;L\;}_{\mathrm{CLS}}$、$w/o\;{\;L}_{\mathrm{UNI}}$ and ${w/o\;L}_{\mathrm{MOD}}$). The results are shown in Fig. 5. Figure 5 presents the results. Substituting AGCN with GAT led to a significant performance drop, which demonstrates the original structural encoder’s superiority in capturing fine-grained molecular features. Replacing the cross-attention network with feature averaging caused a consistent decrease in average performance, thus emphasizing the cross-modal feature fusion strategy’s effectiveness. Performance degradation occurred when removing BDA or any of its subcomponents. The full module enhanced the learned representations’ discriminative power and improved overall prediction accuracy. Its subcomponents, which dynamically adjust class weights, played a crucial role in boosting minority class recognition. These findings collectively verify the proposed modules’ design rationality and synergistic advantages, providing empirical support for further model architecture optimization.

More detailed experimental results and analyses are presented in Supplementary Figs S1–S3, available at supplementary material  *Bioinformatics Advances* online.

## 5 Conclusions

We presented ZSCAN-DDIE, a zero-shot learning framework that combines attention-based graph convolutional networks with a biomedical pre-trained language model to detect previously unidentified DDIEs. By utilizing a cross-attention mechanism, the model correlates molecular substructures with semantic cues derived from biomedical texts, enabling interpretable and fine-grained predictions. Additionally, the bimodal dynamic alignment (BDA) loss module, enhanced by a stage-adaptive loss-switching strategy, effectively reduces modality bias and class imbalance. Experimental results averaged over five independent runs indicate that ZSCAN-DDIE consistently surpasses baseline models in both conventional and generalized zero-shot scenarios, demonstrating superior accuracy, robustness, and interpretability. Attention-based visualizations reveal that ZSCAN-DDIE can identify key functional groups associated with specific DDIEs, providing insights into pharmacological mechanisms and improving clinical decision support. However, this enhanced interpretability is accompanied by increased computational complexity. The integration of cross-attention mechanisms and dual-modality alignment demands additional memory and computation resources, which may restrict scalability in large-scale or real-time clinical applications. Future work will concentrate on optimizing model efficiency and validating the discovery capability of ZSCAN-DDIE on newly emerging drug-drug interactions through external pharmacovigilance databases, newly released DrugBank versions, and prospective biological experiments. Such validation would further demonstrate the practical value of the proposed framework for discovering previously unknown DDIEs and supporting clinical drug safety assessment.

## Supplementary Material

vbag243_Supplementary_Data

## Data Availability

The source code and data are available at https://github.com/GSX-0429/ZSCAN-DDIE.The authors declare that all data and code supporting the findings of this study are publicly available and can be accessed at https://github.com/GSX-0429/ZSCAN-DDIE.
