# Supplementary material for: ZSCAN-DDIE: an interpretable zero-shot learning method for the prediction of drug-drug interaction events using biomedical text
Source: Bioinform Adv. 2026 Aug 19;6(1):vbag243. doi: 10.1093/bioadv/vbag243 (PMC13570678; doi:10.1093/bioadv/vbag243)
Supplement: Supplementary file 1 [file vbag243_supplementary_data.docx]

# Related work

## Zero-shot learning

As a subfield of transfer learning, zero-shot learning (ZSL) aims to enable models to recognize classes that are not observed during training. Originating in the field of computer vision, the theoretical framework of ZSL is characterized by two core components. First, it constructs a shared semantic space that allows both seen and unseen class representations to be mapped into a unified embedding. Second, it defines a compatibility function to bridge the knowledge transfer across classes, by measuring the similarity between input features and semantic descriptions to facilitate accurate classification of previously unseen categories [1] This approach draws inspiration from human cognitive mechanisms. Humans are capable of recognizing novel objects by transferring semantic knowledge from known concepts. For example, given the description "a zebra has the shape of a horse but is covered with stripes," one can recognize a zebra even without prior exposure, by combining the known visual concept of a horse with the semantic understanding of stripes [2]. In algorithmic terms, this cognitive ability is modeled by introducing auxiliary semantic information to construct a unified representational space. Modern ZSL methods generally adopt semantic spaces as their core framework [3], typically designed along three key dimensions: (1) Semantic Embedding Dimension—transforming class labels into computable semantic vectors; (2) Feature Projection Dimension—establishing a mapping between visual features and semantic representations; and (3) Space Inclusiveness—ensuring the embedding space encompasses both seen and unseen classes. Through this structured design, models can perform cross-class knowledge transfer under a unified semantic framework.

Recent advances in ZSL focus on innovations in compatibility modeling paradigms, which aim to align visual features with the semantic space through refined matching strategies. Notably, methods such as DeViSE [4], ALE [5], and Cacheux et al. [6] utilize ranking-based optimization strategies, employing margin-based losses to control the relative distance between matched and unmatched pairs. In contrast, probabilistic optimization paradigms, as introduced in DUET [7] and by Sumbul et al. [8], directly maximize the likelihood of correct class matches using cross-entropy loss. To support these modeling mechanisms, three primary types of semantic information sources have emerged: (1) Class-name embedding-based methods, such as those by Frome et al. [4] and Li et al. [9], which rely on pre-trained word embeddings to capture semantic relationships among class names; (2) Attribute-based methods, exemplified by Lampert et al. [10] and Al-Halah et al. [11], which use handcrafted or learned attribute sets to provide fine-grained semantic representations; and (3) Text-description-based methods, such as those proposed by Reed et al. [12] and Zhu et al. [13], which leverage contextual information from natural language descriptions to enhance the expressiveness of semantic embeddings, ultimately enabling transferable recognition in a shared semantic space.

## Drug-drug interaction events prediction

In the realm of Drug-Drug Interaction Event (DDIE) prediction research, existing studies can be broadly classified into three primary approaches: deep neural network (DNN), graph neural network (GNN), and tensor factorization method.

DNN-based methods capitalize on deep neural networks' potent feature extraction abilities for DDIE prediction. As deep learning has advanced, prediction accuracy has surged remarkably. In 2019, Lee et al. [14] presented a model merging three drug feature types as DNN input for DDIE prediction. To gain deeper insights into various drug-drug interactions' (DDIs) pharmacological effects, the model utilized multiple autoencoders alongside deep feedforward neural networks. Also in 2019, Hou et al. [15] brought forth a deep neural network architecture constructed with TensorFlow-GPU, leveraging drug data from DrugBank. Their model pinpointed 80 distinct DDIE types from 4,432 drug features. In 2020, Deng et al. [16] scrutinized DrugBank's DDIE data, centering on 65 drug interaction event categories across 572 drugs and 74,528 interaction associations. They amalgamated multiple data sources, employing chemical substructures, drug targets, enzymes, and pathways as features to compute similarities. Subsequently, a multi-channel deep neural network was established to learn drug multimodal representations for DDIE prediction. More recently, in 2022, Shao et al. [17] introduced TBPM-DDIE, a Transformer-based DDIE prediction model. TBPM-DDIE generates latent representations encoding drugs' structural and semantic information, which, when combined with other drug features, serve as input to the interaction event classifier. The authors utilized the ChEMBL24 dataset, comprising 1,722,297 unlabeled drug SMILES strings, for Transformer pre-training. Post-preprocessing, the raw SMILES strings were transformed into latent vectors capturing the drugs' underlying structural and semantic features.

GNN-based approaches have been widely adopted in DDI prediction due to their ability to effectively model graph-structured data. In 2021, Lyu et al. [18] proposed a multimodal framework based on deep neural networks for DDIE prediction. Their model leveraged GNNs on drug knowledge graphs to capture both topological and semantic relationships among drugs. By constructing a multimodal neural network, they jointly represented structural information of drugs while incorporating heterogeneous features from different modalities. This design enhanced feature complementarity and improved predictive performance. In 2023, Xiong et al. [19] introduced MRCGNN, a multi-relational contrastive learning-based GNN model. Their method applied GNNs on a multi-relational DDIE graph and integrated structural features extracted from molecular graphs of drugs. By combining structural and interaction information, MRCGNN addressed the challenge of predicting rare DDIEs, which often suffer from insufficient labeled instances in traditional GNN models.

Tensor factorization-based methods model drug-drug interactions as high-dimensional tensors and utilize tensor decomposition techniques to uncover complex drug interaction patterns. In 2017, Jin et al. [20] proposed a tensor-factorization-based multi-task binary prediction model to forecast adverse drug-drug interaction types. By simultaneously identifying multiple potential interaction types between drug pairs, their method effectively captured task relatedness, substantially enhancing prediction performance. Moreover, it alleviated the confounding effects of covariates in clinical observational data, further boosting predictive accuracy. Feng et al. [21] introduced the DeepMDDI model, which adopts a tensor-like decoder to simulate drug interactions. This model can determine whether specific unannotated drug pairs in a DDI network are likely to exhibit one or more pharmacological effects and can also predict potential DDIs between existing drugs and new compounds.

In addition, the prediction of drug-drug interactions (DDIs) suffers from the lack of a universally accepted gold-standard dataset. Nevertheless, many existing methods have adopted the dataset introduced by Deng et al. [16] for experimentation and benchmarking. In their DDIMDL approach, a major challenge arises from the limited number of labeled training samples for DDIEs. Therefore, constructing larger and more comprehensive datasets for drug interaction events is of significant importance. The extreme imbalance between the small number of known (positive) interactions and the vast number of potential but unknown (negative) interactions further exacerbate the problem of data imbalance. To address the issue of data scarcity, Deng et al. [22] proposed META-DDIE in 2022, a few-shot learning framework based on their earlier work, DDIMDL. Given that many rare DDIE types are represented by only a handful of instances, the authors employed few-shot learning techniques to generalize from limited labeled data and enable effective classification. META-DDIE uses a DDI representation module and a DDI comparison module to learn from both the chemical structure of drugs and the topological structure of DDI networks to predict interaction events. In 2024, Zhang et al. [23] introduced FSRM-DDIE, a novel few-shot DDIE prediction model designed to further alleviate the impact of data sparsity. Their metric-based meta-learning framework employs a feature extractor that integrates a graph neural network, autoencoder, and Siamese network to effectively learn features for DDIE prediction.

Furthermore, DDI prediction is hindered by the absence of a universally accepted gold-standard dataset. Despite this, numerous existing methods have utilized the dataset presented by Deng et al. [16] for experimentation and benchmarking. In their DDIMDL approach, a key challenge stems from the limited number of labeled training samples for DDIEs. Hence, constructing larger and more comprehensive drug interaction event datasets holds great significance. The extreme imbalance between the scant known (positive) interactions and the vast potential but unknown (negative) interactions further intensify the data imbalance problem. To combat data scarcity, in 2022, Deng et al. [22] proposed META-DDIE, a few-shot learning framework building on their prior DDIMDL work. Given that numerous rare DDIE types are represented by only a few instances, the authors utilized few-shot learning techniques to generalize from limited labeled data and facilitate effective classification. META-DDIE employs a DDI representation module and a DDI comparison module to learn from both drug chemical structures and DDI network topological structures for interaction event prediction. In 2024, Zhang et al. [23] introduced FSRM-DDIE, a novel few-shot DDIE prediction model aiming to further mitigate data sparsity's impact. Its metric-based meta-learning framework features a feature extractor that integrates a graph neural network, autoencoder, and Siamese network to effectively learn DDIE prediction features.

However, few studies have specifically targeted the zero-shot DDIE (ZS-DDIE) prediction task. Most existing approaches struggle to generalize to unseen interaction types as they fail to encode semantically meaningful and transferable DDIE representations. Notably, the 3DGT-DDI [24] model exhibited promising zero-shot capabilities by using the pre-trained SciBERT language model to derive DDIE representations from drug textual descriptions. Additionally, Wang et al. [25] proposed ZeroDDI, which addresses ZS-DDIE prediction's representation learning and class imbalance challenges through enhanced biomedical semantic learning and a cross-modal matching strategy.

Despite these advancements, the current ZS-DDIE prediction task still faces several challenges. First, existing models lack interpretability. They fail to reveal the underlying mechanisms at the molecular level. This limits their usefulness in clinical and research settings. Second, the distribution of DDIE categories is highly imbalanced. Mainstream models struggle to recognize rare or zero-shot classes. This severely affects the overall prediction performance and generalization ability.

# Experiment settings

Our method is implemented using Python 3.9 and the PyTorch 1.11.0 framework. All experiments are conducted on a platform running Ubuntu 20.04, equipped with an Intel(R) Xeon(R) Gold 6430 processor and an NVIDIA L20 GPU. The model is built upon PyTorch-Geometric 2.1.0, RDKit 2024.3.5, and DeepChem 2.8.0. For the language representation component, we adopt BioBERT Base v1.2. The detailed hyperparameter settings for ZSCAN-DDIE are summarized in Table S1.

Table S1. Experimental setting

| Hyper-parameter | Value |
| --- | --- |
| Batch size | 64 |
| Number of training epochs | 50 |
| Learning rate for Adam optimizer | 0.0001 |
| Dropout ratio | 0.5 |
| Number of layers of AGCN | 2 |
| Dimension of substructure embeddings | 300 |
| Dimension of drug pair representations | 256 |
| Number of substructure representations | 30 |
| Alignment loss weight $\lambda_{1}$ | 1 |
| Distribution loss weight $\lambda_{2}$ | 0.7 |

# Case study

## The interpretability of ZSCAN-DDIE

ZSCAN-DDIE was capable of extracting substructural information from molecules and capturing key features related to DDIEs. Based on attention weights, we highlighted important nodes within the molecular graphs, which were identified by the model as crucial molecular substructures. These nodes potentially reflect underlying mechanisms of DDIEs. To evaluate the interpretability of ZSCAN-DDIE, we randomly selected two drug combinations from the test set that were correctly predicted: Ergometrine–Isoprenaline and Meperidine–Butalbital. Using the open-source RDKit molecular visualization toolkit in combination with attention weight analysis, we visualized the crucial molecular substructures, as shown in Figure S1.

Ergometrine, as shown in Figure S1 (b), an ergot alkaloid derivative, exerts vasoconstrictive effects on peripheral blood vessels and acts as an α-adrenergic receptor antagonist. Isoprenaline, as shown in Figure S1 (a), a non-selective β-adrenergic receptor agonist, is known for its bronchodilatory effects, ability to increase heart rate, and enhance myocardial contractility. Pharmacological studies have shown that the concomitant use of ergot derivatives and adrenergic agonists with hypertensive potential may lead to elevated cardiac output and blood pressure due to the vasoconstrictive properties of the ergot compounds [26]. Structurally, ergometrine possesses a tetracyclic ergoline skeleton—comprising an indole ring fused to a multicyclic system—which resembles serotonin (5-HT) in both shape and spatial distribution. This similarity allows ergometrine to bind to 5-HT₂ receptors and α-adrenergic receptors.

Meperidine, as shown in Figure S1 (d), is a synthetic phenylpiperidine-class opioid receptor agonist with analgesic and sedative properties. Butalbital, as shown in Figure S1 (c), a barbiturate sedative, is used to relieve anxiety and muscle tension. The synergistic effects of these two drugs on the central nervous system may exacerbate adverse reactions such as respiratory depression or coma [27]. Furthermore, barbiturates may promote the formation of toxic metabolites from meperidine, possibly due to the presence of the 1,3-diazinane-2,4,6-trione moiety in their molecular structure.

The visualization results demonstrated that the ZSCAN-DDIE model was able to effectively identify key pharmacophore structures in the ZS-DDIE prediction task. These findings confirm the reliability of ZSCAN-DDIE in identifying functionally significant substructures. More importantly, the interpretability offered by attention-based visualization provides researchers and clinicians with a tangible means of inspecting the model’s reasoning, thereby bridging the gap between computational predictions and pharmacological insights into DDIE mechanisms.

Figure S1. Examples of DDIE textual descriptions and the corresponding visualization of molecular structures of drug pairs.

## Analysis of the association between events

To explore potential correlations between different events, we applied t-SNE to visualize the distribution of event features, as shown in Figure S2, and observed that certain events were closely related to others. We represented each event using the average of the feature vectors of its corresponding drug pair. After computing the Euclidean distances between event features, we identified three pairs of the most similar events (marked with triangles in Figure S2), and selected one representative pair for further analysis. Specifically, events 30 and 38 were found to be close in the feature space and both were associated with arrhythmia-related interactions.

In event 30, Drug1 (e.g., calcium acetate) was likely to enhance the proarrhythmic and cardiotoxic effects of Drug2 (e.g., digitoxin). In the nearby event 38, Drug1 (e.g., edrophonium) was likely to augment the atrioventricular block and arrhythmogenic effects of Drug2 (e.g., digoxin). These findings suggested that the representations learned by the ZSCAN-DDIE model effectively captured the relational structure among DDIEs, thereby facilitating further investigation into inter-event associations and their underlying mechanisms.

Figure S2. Visualization of event features by t-SNE. The triangles represent the locations of three pairs of events with comparable distances in feature space, and the blue dots show the distribution of all events in feature space.

# Hyper-parameter sensitivity analysis

We further investigate the sensitivity of model performance to two key hyperparameters: the number of AGCN layers L and the number of multi-head attention heads H in the CAN module. While keeping all other hyperparameters fixed, we vary the number of AGCN layers as $L\in\{2,3,4\}$ and the number of attention heads as $H\in\{2,4,8,16\}$. All models are evaluated under identical experimental settings, and the corresponding results are reported in Figure S3. Overall, the model achieves the best performance when 𝐿=2 and H=4.

From the perspective of AGCN depth, the model exhibits optimal overall performance at L=2. As the number of layers increases to 4, the ${ACC}^{H}$metric shows varying degrees of degradation. This observation suggests that a moderate increase in graph convolutional depth facilitates the modeling of higher-order structural dependencies within drug molecular graphs. However, excessively deep AGCN architectures are prone to over-smoothing, which diminishes the discriminative capacity of node representations.

With respect to the number of attention heads in CAN, model performance demonstrates a clear non-monotonic trend across different values of H. In particular, H=4 consistently yields stable and superior results across most AGCN configurations. In contrast, an insufficient number of attention heads constrains the expressiveness of cross-modal feature interactions, whereas an excessively large number of heads may lead to overly fragmented attention distributions, introducing redundant information and ultimately degrading the model’s generalization ability.

Figure S3. Parameter sensitivity analysis.

# References

[1]	Shah, N., C. Jacopo, and B. Alessio Del, *Semantically Grounded Visual Embeddings for Zero-Shot Learning.* 2022 IEEE/CVF Conference on Computer Vision and Pattern Recognition Workshops (CVPRW), 2022.

[2]	Fu, Z., et al. *Zero-shot object recognition by semantic manifold distance*. in *2015 IEEE Conference on Computer Vision and Pattern Recognition (CVPR)*. 2015.

[3]	Wang, W., et al., *A Survey of Zero-Shot Learning.* ACM Transactions on Intelligent Systems and Technology (TIST), 2019. **10**(2): p. 1-37.

[4]	Andrea, F., et al., *DeViSE: A Deep Visual-Semantic Embedding Model.* Neural Information Processing Systems, 2013.

[5]	Akata, Z., et al., *Label-Embedding for Image Classification.* IEEE Trans Pattern Anal Mach Intell, 2016. **38**(7): p. 1425-38.

[6]	Yannick Le, C., B. Hervé Le, and C. Michel, *Modeling Inter and Intra-Class Relations in the Triplet Loss for Zero-Shot Learning.* 2021 IEEE/CVF International Conference on Computer Vision (ICCV), 2019.

[7]	Zhuo, C., et al., *DUET: Cross-Modal Semantic Grounding for Contrastive Zero-Shot Learning.* Proceedings of the AAAI Conference on Artificial Intelligence, 2023.

[8]	Gencer, S., C.R. Gokberk, and A. Selim, *Fine-Grained Object Recognition and Zero-Shot Learning in Remote Sensing Imagery.* IEEE Transactions on Geoscience and Remote Sensing, 2018. **56**(2): p. 770-779.

[9]	Li, X., et al. *Zero-shot Image Tagging by Hierarchical Semantic Embedding*. in *Research and Development in Information Retrieval*. 2015.

[10]	Lampert, C.H., H. Nickisch, and S. Harmeling, *Attribute-based classification for zero-shot visual object categorization.* IEEE Trans Pattern Anal Mach Intell, 2014. **36**(3): p. 453-65.

[11]	Al-Halah, Z. and R. Stiefelhagen. *Automatic Discovery, Association Estimation and Learning of Semantic Attributes for a Thousand Categories*. in *2017 IEEE Conference on Computer Vision and Pattern Recognition (CVPR)*. 2017.

[12]	Reed, S., et al. *Learning Deep Representations of Fine-Grained Visual Descriptions*. in *2016 IEEE Conference on Computer Vision and Pattern Recognition (CVPR)*. 2016.

[13]	Zhu, Y., et al. *A Generative Adversarial Approach for Zero-Shot Learning from Noisy Texts*. in *2018 IEEE/CVF Conference on Computer Vision and Pattern Recognition*. 2018.

[14]	Lee, G., C. Park, and J. Ahn, *Novel deep learning model for more accurate prediction of drug-drug interaction effects.* BMC Bioinformatics, 2019. **20**(1): p. 415.

[15]	Hou, X., J. You, and P. Hu. *Predicting Drug-Drug Interactions Using Deep Neural Network*. in *Machine Learning and Computing*. 2019.

[16]	Deng, Y., et al., *A multimodal deep learning framework for predicting drug-drug interaction events.* Bioinformatics, 2020. **36**(15): p. 4316-4322.

[17]	Zhenjie, S., Q. Ying, and D. Liang, *TBPM-DDIE: Transformer Based Pretrained Method for predicting Drug-Drug Interactions Events.* 2022 IEEE 46th Annual Computers, Software, and Applications Conference (COMPSAC), 2022.

[18]	Lyu, T., et al., *MDNN: A Multimodal Deep Neural Network for Predicting Drug-Drug Interaction Events.* International Joint Conferences on Artificial Intelligence Organization, 2021.

[19]	Zhankun, X., et al., *Multi-Relational Contrastive Learning Graph Neural Network for Drug-Drug Interaction Event Prediction.* Proceedings of the AAAI Conference on Artificial Intelligence, 2023.

[20]	Jin, S., et al., *A general hypergraph learning algorithm for drug multi-task predictions in micro-to-macro biomedical networks.* PLoS Comput Biol, 2023. **19**(11): p. e1011597.

[21]	Feng, Y.H., et al., *deepMDDI: A deep graph convolutional network framework for multi-label prediction of drug-drug interactions.* Anal Biochem, 2022. **646**: p. 114631.

[22]	Deng, Y., et al., *META-DDIE: predicting drug-drug interaction events with few-shot learning.* Brief Bioinform, 2022. **23**(1).

[23]	Zhang, L., et al., *FSRM-DDIE :  few-shot learning methods based on relation metrics for the prediction of drug-drug interaction events.* Applied Intelligence, 2024(prepublish): p. 1-14.

[24]	Lv, Q., et al., *3D graph neural network with few-shot learning for predicting drug-drug interactions in scaffold-based cold start scenario.* Neural Netw, 2023. **165**: p. 94-105.

[25]	Wang, Z., et al., *ZeroDDI: a zero-shot drug-drug interaction event prediction method with semantic enhanced learning and dual-modal uniform alignment.* arXiv preprint arXiv:2407.00891, 2024.

[26]	Rodman, R.E., et al., *Ischemic colitis secondary to ergotamine use: a case study.* Case Rep Gastroenterol, 2011. **5**(1): p. 1-4.

[27]	Harbell, M.W., et al., *Anesthetic Considerations for Patients on Psychotropic Drug Therapies.* Neurol Int, 2021. **13**(4): p. 640-658.
